# 3D Printing of Customizable Phantoms to Replace Cadaveric Models in Upper Extremity Surgical Residency Training

**DOI:** 10.3390/ma15020694

**Published:** 2022-01-17

**Authors:** Elisha Raeker-Jordan, Miguel Martinez, Kenji Shimada

**Affiliations:** 1 Department of Mechanical Engineering, Carnegie Mellon University, 5000 Forbes Avenue, Pittsburgh, PA 15213, USA; miguelm1@andrew.cmu.edu (M.M.); shimada@cmu.edu (K.S.); 2 Department of Biomedical Engineering, Carnegie Mellon University, 5000 Forbes Avenue, Pittsburgh, PA 15213, USA

**Keywords:** 3D printing, phantom, residency training, upper extremity, personalized medicine

## Abstract

Medical phantoms are commonly used for training and skill demonstration of surgical procedures without exposing a patient to unnecessary risk. The discrimination of these tissues is critical to the ability of young orthopedic surgical trainees to identify patient injuries and properly manipulate surrounding tissues into healing-compliant positions. Most commercial phantoms lack anatomical specificity and use materials that inadequately attempt to mimic human tissue characteristics. This paper covers the manufacturing methods used to create novel, higher fidelity surgical training phantoms. We utilize medical scans and 3D printing techniques to create upper extremity phantoms that replicate both osseous and synovial geometries. These phantoms are undergoing validation through OSATS training of surgical residents under the guidance of attendings and chief residents. Twenty upper extremity phantoms with distal radius fracture were placed into traction and reduced by first- and second-year surgical residency students as part of their upper extremity triage training. Trainees reported uniform support for the training, enjoying the active learning exercise and expressing willingness for participation in future trials. Trainees successfully completed the reduction procedure utilizing tactile stimuli and prior lecture knowledge, showing the viability of synthetic phantoms to be used in lieu of traditional cadaveric models.

## 1. Introduction

Introducing anatomical features and surgical practices in medical education often relies on cadaveric tissue samples to provide hands-on experience; however, they are not a perfect substitute for live tissue [1]. Tissue degradation occurs shortly after death, and preservative chemicals also introduce changes to the mechanical properties of the sample. Cadaveric models are often single-use due to added degradation from the training procedure, and large-volume training remains limited by high procurement and on-site refrigerated storage costs [2]. Medical phantoms utilize synthetic materials to replicate the hard and soft tissue mechanical behavior of live persons, allowing for customized models displaying specific features required for the execution of surgical techniques [1,3]. For the orthopedic upper extremity phantoms presented here, perfectly copying all human anatomy would be unnecessary and wasteful; only the anatomical features relevant to a specified training procedure should be included. This simplifies manufacturing and improves stimuli recognition and retention in novice trainees [3], including visual, tactile, and auditory sensations used to guide the application of care [1,4].

In addition to the specificity offered by medical phantoms, cadavers can vary greatly in tissue property and size. Manufacturing training phantoms offers reproducibility, further standardizing the educational environment and reinforcing skill acquisition [5]. Variations across multiple training sessions could result in students missing relevant features or misidentifying cues that should not be present, resulting in confirmation bias which can blunt long-term performance [6]. “Practice is a positive constructive process if and only if that practice is an adequate substitute for the true task” [1]: poor practice produces poor performance. Patient care will always present inherent challenges but enabling residents to develop these skills earlier improves their ability to acquire future skills, improving care and limiting potential unintended harm [7,8,9].

**Figure 1:**
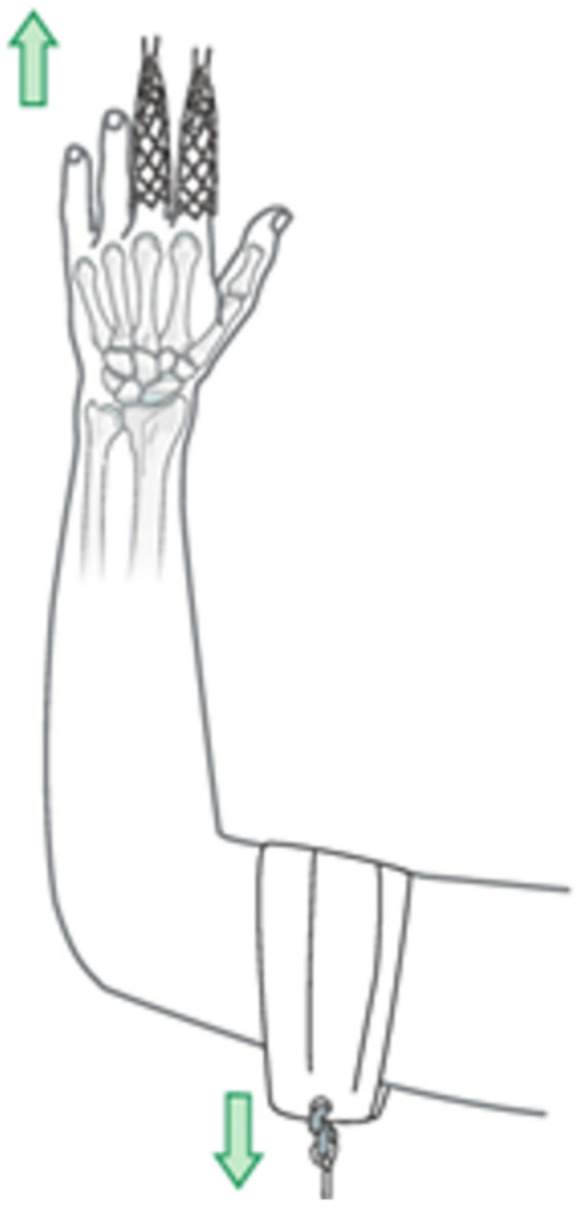
Illustration of a commonly used technique, the finger-trap, to induce traction in a distal radius fracture (DRF) patient.

We designed an upper extremity phantom to address orthopedic management of distal radius fractures (DRF), a procedure typically utilizing cadaveric models. In most cases of DRF, initial treatment involves reduction and splinting, as shown in Figure 1. Any model used to demonstrate this procedure must exhibit behavior sufficient to replicate that of a live patient. We do not know of any low-cost commercial DRF models with sufficient features, as described by practicing surgeons, to train orthopedic residency students [1,10]. Existing models share many common characteristics such as oversimplified soft tissues, a narrow focus on distal behavior without considering interoperative proximal features, and generic casting or splinting. While high-profile manufacturers SawBone and Blue Phantom do produce phantoms targeting a single training action, models remain too generic and must be further altered to exhibit the behavior required to train DRF specifically [11]. The range from low-end to top-spec phantoms is quite wide, with some costing hundreds of dollars and others multiple thousands, while remaining inadequate for their intended purpose without significant modifications [1,12].

We wished to improve upon these attempts by working directly with surgeons to integrate clinically important features, while utilizing low-cost materials to maintain advantages over both commercial phantom and cadaveric models [2,13]. We utilized 3D printing and rapid manufacturing to iterate model features with high dimensional accuracy [14] without relying on injection molding or machining components. The cost of materials for our DRF phantom is less than $80 per model. This ignores machine costs, as 3D printers can vary widely in price. While this paper will focus exclusively on designing a DRF model, the proposed materials and manufacturing processes would be applicable to other orthopedic phantoms, necessitating only a change in the initial anatomical geometry.

## 2. Materials and Methods

For our models to exhibit the same sensory feedback surgeons expect from true fractures, we captured and translated the mechanical requirements of DRF reduction into phantom components: (1) a periosteum-like sheath on the bones to improve cartilaginous joint behavior [1], (2) better replicate the behavior and difficulty of manipulating a real patient by creating an entire arm from the phalanges to the proximal humerus [1,15], (3) incorporate the use of radiopaque elements to allow for medical imaging of surgical accuracy [1], and (4) an opaque covering to mimic skin and restrict visual cues from the phantom’s internal structure. Below we describe the required materials and manufacturing methods used to create these phantom components.

### 2.1. Periosteum

**Figure 2:**
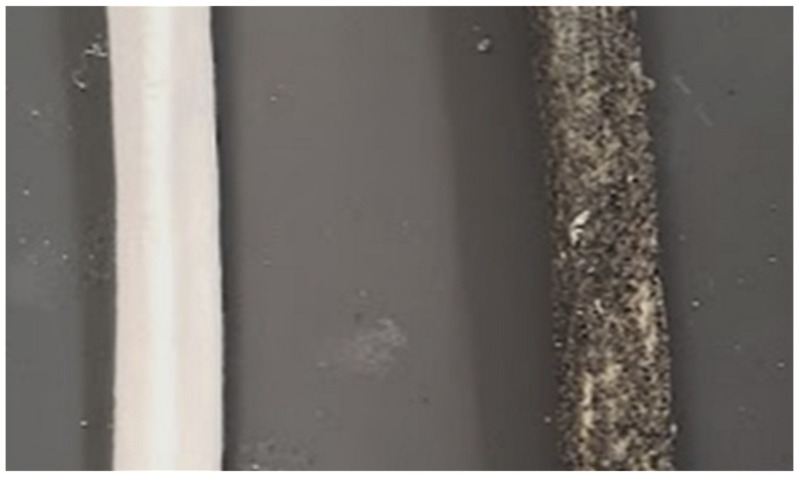
Photograph showing our periosteum-coated phantom bone segment (**right**) beside to a bare 3D print (**left**). Four individually cured layers produce a smooth sheath, improving behavior at joints, and controllable radiopacity.

To create this membrane and other capsuloligamentous structures, Holden’s HX-80 latex (Holden’s Latex, Macungie, USA) was chosen. It has the consistency of latex paint, allowing for controlled application in thin layers, with sufficient viscosity to support mixing additives in suspension. Figure 2 shows this coating with radiopaque particles in suspension adhered to the phantom bone. The coating also provides light structural support in the wrist, acting like cartilage between carpal bones and creating a more lifelike response. This reduced the need for additional support rods connecting smaller bones during casting, which may have interfered with tactile feedback. Material cost for the entire phantom periosteum is less than $3.

### 2.2. Opaque Skin Covering

**Figure 3:**
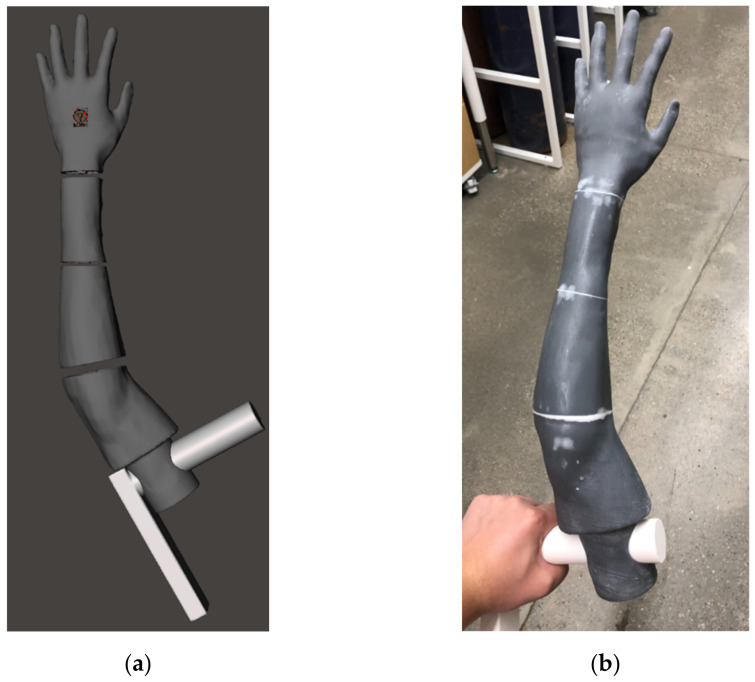
(**a**) Mirrored copy of phantom arm geometry (gray), with grip (white), in Meshmixer. (**b**) Skin covering overmold printed in parts with FormLabs resin.

To restrict the naked visual feedback of the phantom bones inside the clear gelatin flesh, we created an opaque skin covering. This covering fits the complex 3D contours of the phantom model, adding to the fidelity of the model by creating the realistic appearance of skin. We took the arm model shape and printed it directly to create the overmold in Figure 3a, whereas before this asset was used to create a cavity for the casting mold of the phantom. Dyed rubber is then cured on-to the surface printed with FormLabs resin (FormLabs, Somerville, MA, USA) shown in Figure 3b to create, in essence, a glove for the phantom.

**Figure 4:**
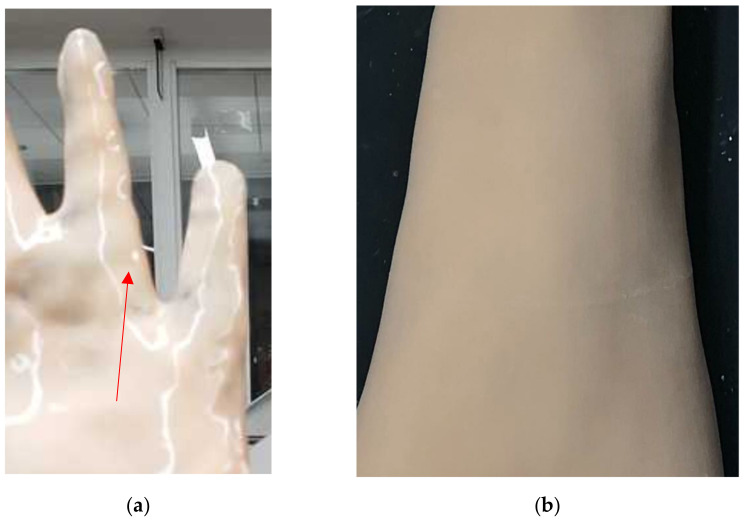
(**a**) DragonSkin surface cured open to the air, arrow points to reflected light indicating a high gloss finish. (**b**) DragonSkin surface cured on FormLabs resin 3D-printed overmold, the matte finish results in a dull but uniform appearance.

By curing DragonSkin 10 two-part silicone rubber (Smooth-On, Macungie, PA, USA) onto the surface of this overmold we could control the thickness of the skin covering as well as the resulting surface texture. A fully enclosed mold would allow for better control over the material thickness, but the rubber will not cure when deprived of oxygen. This necessitates the careful application of the two-part compound onto the outer surface of our overmold. We then encountered an aesthetic issue with the rubber: the surface of the rubber in direct contact with the air produces a glossy surface on the DragonSkin (Smooth-On, Macungie, PA, USA) shown in Figure 4a, but the rubber cured against another solid surface produces a matte texture and reduces the intensity reflected light seen in Figure 4b.

This matte surface appears closer to human skin, adding realism and believability to the target tactile stimuli. This added fidelity reduces friction when students transition to practicing with real patients later in residency [13]. To produce this matte finish on the outside of the skin covering, the arm model was mirrored in Meshmixer (Autodesk, San Rafael, CA, USA), a freely available mesh manipulation software [14], to produce an identical left-arm copy prior to printing. The rubber is applied to the surface of the left-arm overmold and the cured skin is inverted when removed. This creates a surface that will perfectly conform to our right-arm phantom model. Material cost for one phantom’s skin covering is approximately $10.

### 2.3. Extended Phantom Skeleton

**Figure 5:**
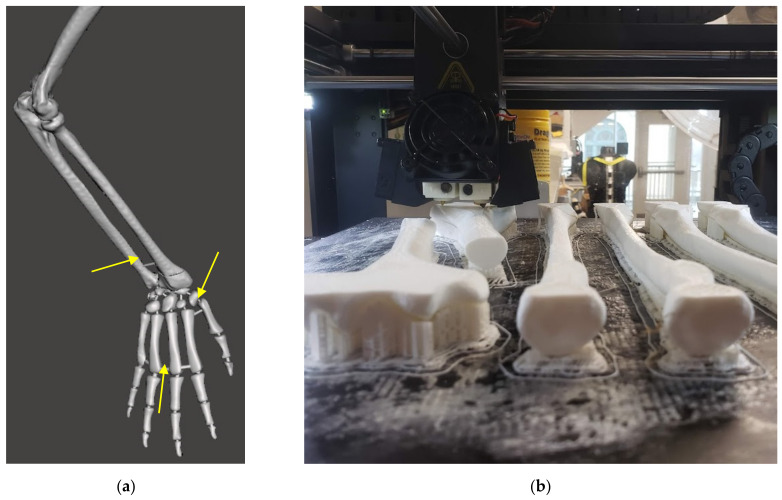
(**a**) Photograph showing the modified bone model in Meshmixer. Individual bones are fixed to one another with small pins, examples identified with yellow arrows. The phantom created for this study also incorporates a DRF modification, circled in red. (**b**) Sample long-bones of the phantom skeleton being printed with the Raise3D FDM machine.

The DRF phantom described in this paper was an expansion on previous work developing a carpal fracture pinning model [15]. This phantom included all carpals and metacarpals but was truncated mid-forearm, removing the interaction of the radius and ulna at the elbow. To effectively replicate gross arm movement with interoperable joint behavior between the wrist and elbow, the same design process was used on a full upper extremity bone model [1]. We 3D-scanned a commercially available synthetic bone model from SawBone [16], creating a 3D polygonal file to set the relative orientations of the hand, forearm, and upper arm. We coordinated with our surgical partners on the phantom pose required to simulate finger-trap traction [1]. Figure 5a shows the posed upper extremity bone model in Meshmixer after scan artifacts and other surface errors were patched. Small pins are added between adjacent bones to maintain their relative positioning during printing, coating, and casting. These pins break, still contained within the periosteum layer and ballistic gelatin, when first manipulating the phantom and do not interfere with further use [1,15].

The modified bone model is then manufactured by fused deposition modeling (FDM) with the Raise3D Pro2 printer series [17], as shown in Figure 5b. Human bones are not solid, and the mesh lattice created by FDM printing closely replicates the cancellous geometry of bone [18]. This bone composition was chosen during our previous work on a percutaneous pinning model to give cortical (outer shell) and cancellous (inner lattice) feedback to surgeons [15]. This not only makes our models splinter and deform like bones, but also drastically reduces infill material and speeds up printing time. The non-invasive surgical operations in this DRF management training would not change if using solid or hollow bones. However, the evolving nature of this model may again require percutaneous pinning or other invasive training, and as such, the bone composition has been maintained. The cost to print bones for one phantom, including wasted support structures, is less than $4.

**Figure 6:**
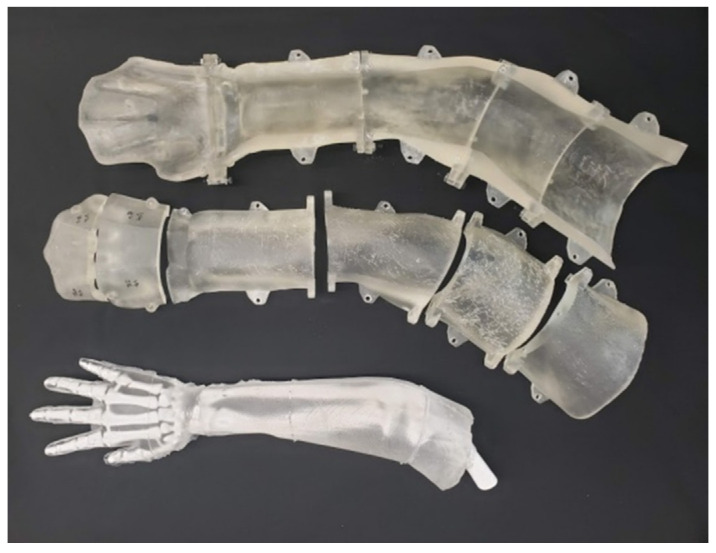
Photograph showing an unskinned phantom arm (**bottom**) beside its multi-piece casting mold (**top**) printed from FormLabs’ SLA resin.

**Figure 7:**
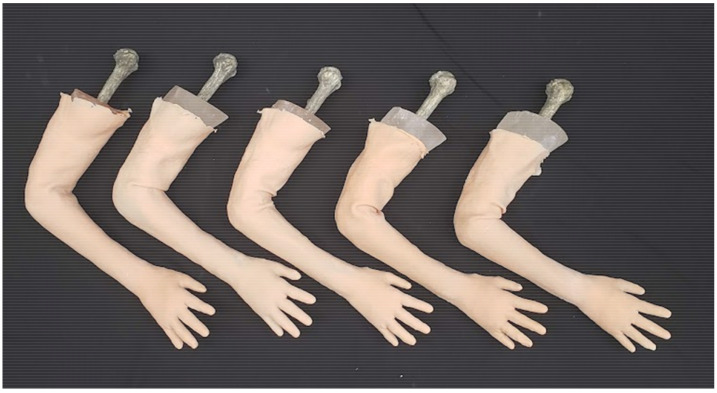
Photograph showing the replicability of full-arm phantoms, with slight variations seen in flesh and skin truncation along the humerus.

**Figure 8:**
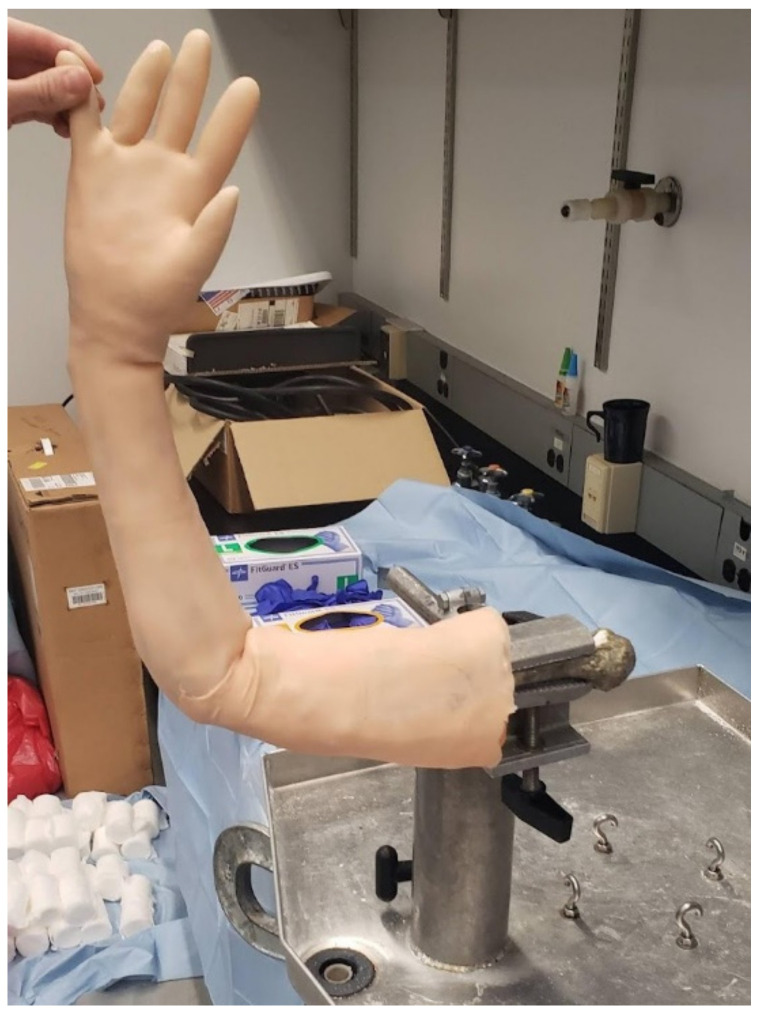
Photograph showing our phantom mounted in a vice to replicate a stationary patient in preparation for inducing traction.

Repeating the procedure for setting the bone pose, we then scanned the arm of a lab member who closely matched the size of the SawBone. This scan was used in Meshmixer to create the mold for casting the bones. After using Boolean operations to create the internal cavity of the mold, we separated it into segments for 3D printing. The bones are cast using ballistic gelatin, which must be heated above 275 °F (135 °C) to melt. The mold pieces are printed using stereolithographic (SLA) FormLabs Form3 resin printers (FormLabs, Somerville, MA, USA), which create smoother and more temperature-resistant prints than those produced by FDM [19]. The pieces are reassembled into the reusable molds shown in Figure 6. The ballistic gelatin required for one phantom costs approximately $60. After the heated gelatin cools, the model can be demolded and covered in our opaque skin, multiple of which are shown in Figure 7. These completed models are sent to our collaborating surgeons for evaluation, as shown in Figure 8, when revising the fracture site or joint pose. If that revision of the model responds appropriately to manipulation, they are used to practice and assess DRF management techniques in residents.

### 2.4. Medical Imaging

**Figure 9:**
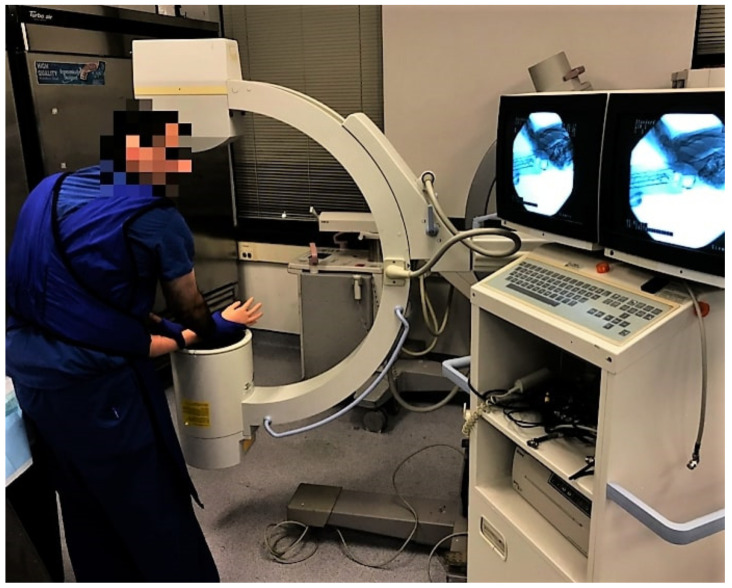
Photograph showing the examination of the phantom using C-arm fluoroscopy.

As part of DRF management techniques, after a patient is placed into a splint or cast, it is required to get a medical scan of the bones to ensure they will heal in the proper orientation. Fluoroscopy is a commonly used X-ray scan to give the surgeon a real-time view of patient anatomy, demonstrated in Figure 9. To see differentiation in tissue with X-ray, those tissues must differ in density with hard tissues (bones), causing more interference than soft tissues (flesh). Our model materials initially created a different relationship, with the ballistic gelatin obfuscating the less dense plastic bones.

**Figure 10:**
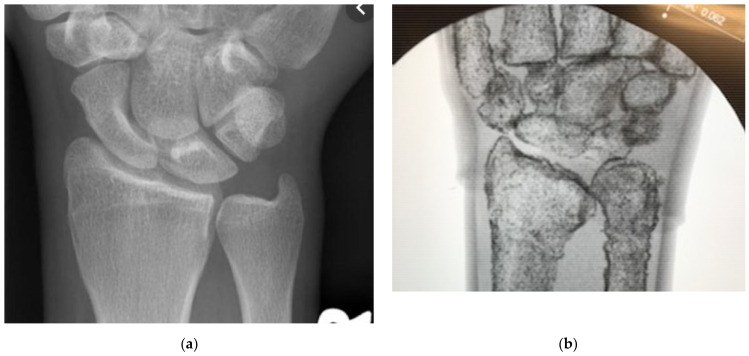
(**a**) Human hand under fluoroscopy to show hard and soft tissue feedback. (**b**) Our DRF phantom under fluoroscopy demonstrating differentiable hard and soft replica tissues [1].

**Figure 11:**
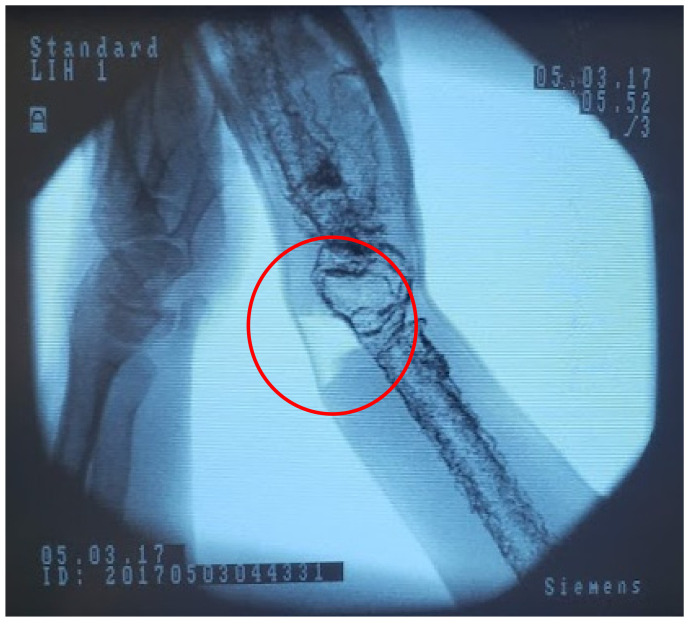
Fluoroscope image of our phantom showing a tear in the ballistic gelatin (circled) after manipulation of the joint.

Rather than change the material composition of the phantom, which would require us to remake portions of the model from the ground up with more expensive components, we chose to leverage the behavior of X-rays and metal to create artificial feedback. We settled on the low-cost option of mixed iron powder with the latex periosteum to raise the apparent density of the material. As the high-density iron would be localized to the bone surface, it would create interference with the X-rays, allowing us to overcome the lack of inherent density in our materials. We mixed batches of latex and iron by increasing the weight fraction of the iron by 5% and then tested them under fluoroscopy to gauge the ease with which constitutive features were shown when compared to live tissues. This comparison is shown statically with fluoroscopic images of a human wrist in Figure 10a and our DRF phantom in Figure 10b. The two provide sufficiently distinct imaging responses while providing the same tactile feedback to differentiate hard and soft tissue features. “On average 40%/weight of iron filings” in four layers of latex was sufficient to visualize the phantom bones [1]. The iron required for a single phantom is under 10 g, an essentially negligible cost. The iron-laden latex maintains the same curing time and conditions, and surgical feedback indicated no differentiation to the tactile response of the overall model. Slight changes, if any, to performance are likely overshadowed by the ballistic gelatin-bulk tissue behavior. Manipulating the fracture while imaging, as shown in Figure 11, allows residents to pair tactile and visual stimuli to create a more holistic understanding of the procedure [1,6,8,9,20,21].

## 3. Results

After iterating the phantom to consistently reproduce the target behavior with our collaborating surgeons, the next step was resident testing to determine its viability with novices. Twenty early (post-graduate years 1 and 2) upper extremity surgical residents, at a mid-size teaching university in the mid-Atlantic region of the United States, performed DRF management on our phantoms across two sessions in the Falls of 2019 and 2021. No in-person study was performed in 2020 due to COVID-19 restrictions. Our surgical collaborators’ Institutional Review Board approved this practical testing with volunteer orthopedic surgery residents.

In the first training session, 2019, four residents participated in the phantom training. In the second training session, 2021, sixteen additional trainees participated in phantom training. Trainees were all in their first or second year of residency, and their familiarity with DRF is primarily classroom experience. We are still processing data from this 2021 study; the following results are those of the Fall 2019 training. The intent of these training sessions was to determine the phantom’s ease of use and ability to supplant cadaveric models.

Residents were assessed via the objective structured assessment of technical skills (OSATS) tool [22]. This metric is commonly used to judge surgical performance and our partners were familiar with its use. Two licensed, fellowship-trained hand surgeons performed the grading for these training sessions. Assessments included grading of DRF “reduction and immobilization processes with a step-by-step checklist of required tasks, the accuracy of fracture reduction, and time to completion” [1], in addition to a written knowledge test on the fundamental concepts of DRF.

The 2019 training results are presented below in Table 1, and the written exam used in both training sessions can be found in Appendix A. Radial inclination and volar tilt refer to two measures of the final bone pose in the model, a measure of how well the fracture will heal [1,3,7,15]. Residents who passed the hands-on portion of the training exercise adhered to the step-by-step surgical instructions more accurately and performed those instructions more quickly. Better performance in the hands-on training with the phantom also predicted higher scores on the written knowledge exam [22]. This shows that those students who were more familiar with the procedure were able to demonstrate this knowledge through accurate manipulation of the training phantom [1].

All residents successfully completed the DRF exercise, albeit with varying degrees of success. All residents responded that the training was helpful and that the phantom contributed to their motivation. Motivation remains one of the crucial variables when assessing effective learning environments, as much of human behavior relies on leveraging personal enjoyment [1,23]. The focus of this research is, at present, not to determine the exact impact on student learning. This will be possible after more years of data where we can analyze the change in student performance after multiple exposures with the model as part of their comprehensive 5-year residency training. The focus of the interactive sessions discussed here is determining the viability of our phantom in displacing the use of cadaveric models. We have shown that our phantom was able to provide the necessary stimuli for the successful application of radial reduction management techniques by novice residents. All required elements of OSATS grading for this procedure were met by the phantom [22], sufficiently showing competition with cadaveric models without a direct comparison.

## 4. Discussion

The work here in creating relevant clinical features in a DRF phantom shows that cadavers are replaceable for early resident skill training. The training was completed successfully for all participants, demonstrating the phantom’s ability to convey required stimuli normally found from live patients or cadaveric models. Interactive training with synthetic phantoms allows for attending surgeons to evaluate resident performance and grade the application of classroom techniques in a safe, practical, and low-cost environment. As well, the residents indicated their strong approval for this type of learning activity, expressing a willingness to repeat the training or participate in future trials. This internal motivation is key to long-term engagement and integration of component skills, contributing more broadly to increased quality of care for patients [3,4,6,24].

### 4.1. Current Limitations

The DRF phantoms in Figure 7 and Figure 8 were not designed to anthropometric standards [25]: 5% female, 50% male, and 95% male. These measurements are used in setting the size ranges of consumer products and using this standardized generality would also make sense to use for medical training. The current phantom size was set early in the design process by scanning a lab member’s arm but could be scaled up or down at many different areas of the manufacturing process depending on the need for pediatric, juvenile, or adult models.

**Figure 12:**
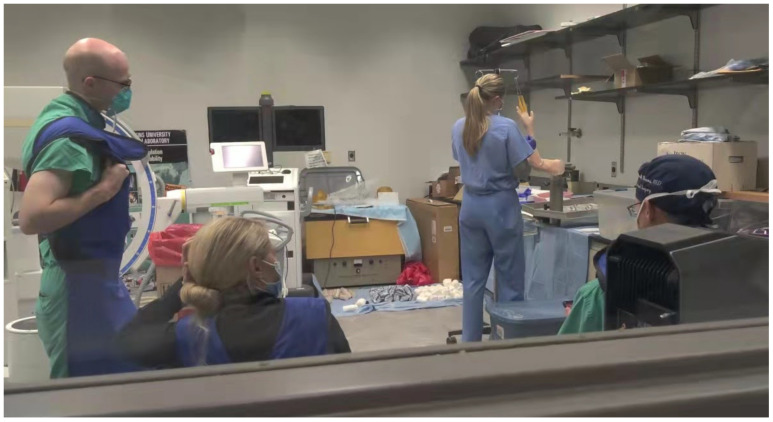
Photograph showing residency student, standing at middle right, practicing with the DRF phantom while under supervision of three surgeons.

Overly aggressive manipulation of the wrist can result in degradation and tearing of the ballistic gelatin, seen in the lighter area circled in Figure 12. This can be seen as a feature, in that potential patient damage can result from using too much force to realign the fracture. Seeing this behavior in models can be used to further demonstrate to residents the potential difficulties of this commonplace procedure. To further the longevity of the phantom we would need to reinforce this area, which may impact the tactile feedback. We will continue weighing the advantages and disadvantages of this material behavior.

The manufacture of the skin coverings is time-consuming and difficult to obtain a high-quality version with even thickness all around the overmold. The individual curing the rubber onto the overmold must continuously move and spin the model to avoid the rubber flowing and pooling before it is fully cured. Aside from an even layer of rubber at the macro scale of the model, local peaks can form on the underside as the material coalesces and begins to drip. Rotating the overmold has proved to be a sufficient solution while the rubber cures, but this takes approximately 45 min of protracted physical effort. This process could be automated using a 6-axis control system to produce perfect rotations of the overmold to ensure even material coverage, however, this would also go against the project ethos of producing a low-cost model. This and other modified manufacturing techniques are being explored throughout the continued development of these phantoms.

We currently rely on the expertise of our surgical collaborators to determine the fitness of each individual phantom. We have found the perceived accuracy of each unit to vary between experts while still providing the relevant information to successfully complete a DRF management within the allowable surgical tolerances. Quality control is somewhat maintained by the high accuracy of the 3D printed components, but additional measures will be required to scale up manufacturability.

### 4.2. Future Work

We are currently processing data from a follow-up study, identical to the testing described above, but with 16 additional novice first- and second-year residency students. Initial results are positive and repeat our previously seen trend of positive student engagement [1].

Manufacturing remains a laborious and time-consuming task as each step is performed by hand, with require multiple curing or cooling phases. A single model requires 20 h to print the bones, 10–15 h of active work preparing and casting the bones interspersed by 30 waiting hours of curing and cooling, in addition to 2 h of active time to create the skin covering, totaling more than 60 h.

Placement of bone within the large mold cavities can be difficult as small joint errors lead to large variations across the length of the forearm. Currently, this requires manual adjustment of the bone position while the gelatin is added in stages. Additional pinning or joint fixation may remedy some of these difficulties.

Cartilage is not extensively replicated in these models aside from the periosteum helping to dampen interactions in joint areas. Latex used for the periosteum coating helps to fill in the empty space between the carpals, and when vulcanized helps perform some of the roles filled by human cartilage. We are currently developing intermediate casting molds to create more defined joint capsules before encasing in ballistic gelatin flesh.

Bone printing and preparation can be done ahead of time without obvious degradation of the materials; however, we have been prone to receiving last-minute revisions, rendering these old models obsolete. With additional printers, ovens, and molds then multiple models can be done at once while others are cooling. As the mold pieces are continually revised while negotiating the model positioning with our surgical partners, injection-molding or other more permanent casting methods are infeasible and prohibitively costly in time and money. We hope with additional validation, we can lock the design of specific orthopedic training procedures and manufacture these phantoms on a larger scaler and with more efficiency.

## 5. Conclusions

This orthopedic surgical training and assessment procedure, traditionally relying on cadaveric models, was successfully completed using synthetic phantoms. Students were able to successfully complete a radial reduction procedure utilizing prior classroom knowledge and enjoyed the opportunity to apply prior classroom knowledge in an active setting. This control over replica anatomical features offered by phantoms cannot be matched in cadaveric samples, enhancing training through repeatability and standardized scoring.

The accuracy and rapid modification of our phantoms is only possible through the fine detailing and rapid production nature of additive manufacturing. The ability to enact incremental feature changes normally too costly or time-consuming to perform with traditional manufacturing techniques gives us an opportunity to address previously unmet or unidentified engineering problems. Working directly with practicing surgeons in a teaching environment gives us the ability to accurately tailor and test our phantoms to provide the truest experience, improving skill acquisition and resident motivation. We are excited to continue the development of this and other high-fidelity models through our partnership.

## Figures and Tables

**Table 1:** Sample OSATS performance scores from 2019 for DRF management resident training performed on our phantom; adapted from [1]. The written exam is found in Appendix A.

	Subject 1	Subject 2	Subject 3	Subject 4
Check List Grader #1 (0–12)	7	5	5	7
Check List Grader #2 (0–12)	6	6	4	8
Radial Inclination (deg)	4	12	3	18
Volar Tilt (deg)	8	0	−2	7
Procedure Time (min)	21.18	36.24	26.34	18.28
Procedure Rating #1 (1–5)	3.14	2.14	2.29	3.57
Procedure Rating #2 (1–5)	3.71	2.00	2.43	3.86
Written Assessment (0–15)	10	5	7	12
Pass/Fail	Pass	Fail	Fail	Pass
Impression of the phantom (Likert 1–5)	5	5	5	5

## Data Availability

Data are contained within the article.

## Appendix A

**Table A1:** Written examination given to trainees following the performance of distal radius fracture management on the phantom, correct responses to multiple choice questions are bolded, adapted from the work in [1].

1. Identify the tendon(s) most likely to rupture as a result of closed reduction and casting of a distal radius fracture.	2. On the figure below, draw how you would measure radial height and inclination (use an x to indicate the radial height and an α to indicate radial inclination).
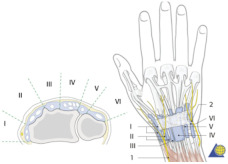
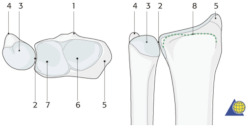
3. What is the acceptable maximum amount of radial shortening for non-operative management of a distal radius fracture (DRF) according to the AAOS clinical practice guidelines? 1 mm 2 mm **3 mm** 4 mm 5 mm	4. What is the maximum accepted dorsal angulation, from neutral, for non-operative management of a DRF according to the AAOS clinical practice guidelines? 0 degrees 5 degrees **10 degrees** 15 degrees
5. What is the maximum acceptable amount of articular displacement for non-operative management of a DRF according to the AAOS clinical practice guidelines? 5 mm 4 mm 3 mm **2 mm** 1 mm No articular displacement can be accepted	6. Which of the following has been associated with loss of reduction and re-displacement following closed reduction of a DRF? Time to initial reduction Severity of initial displacement Hand Dominance Age of the patient **B and D** All of the above
7. Which of the following is a contraindication to isolated closed reduction and percutaneous pinning of a DRF? Intra-articular fracture through the sagittal crista Dorsal comminution with poor bone quality Radial comminution extending proximal to the distal radial ulnar joint in the coronal plane **Volar comminution with an unstable articular fragment**	8. What is considered normal radial inclination? **22–23 degrees** 18–20 degrees 5 degrees for each mm of radial height on AP radiograph 28–30 degrees
9. A polytrauma patient presents to the ED following a motorcycle collision with a distal radius fracture. What is the risk of acute carpal tunnel syndrome in this patient after reduction and splinting? **30 %** 5% 75% 60% <1%	10. A patient has symptoms suggestive of acute carpal tunnel syndrome following a DRF and you are going to the operating room to release their carpal tunnel. Your planned approach to allow for carpal tunnel release may include: Extensile ulnar approach Modified Henry approach only Classic Henry approach with second incision **A and C** All of the above
11. A patient presented to the ED with a DRF from a ground-level fall. The DRF was closed reduced by an on-call resident. After discharge from the ED the patient calls your office with a complaint of worsening pain and progressive loss of sensation in the thumb and index finger. What splinting position could have put this patient at increased risk to develop this complication and what is the incidence of this complication occurring in this setting? Excessive flexion only, approximately 20% Excessive flexion and ulnar deviation, approximately 40% Excessive ulnar deviation only, approximately 10% **Excessive flexion and ulnar deviation, approximately 10%**	12. A 75-year-old woman has a DRF that is closed reduced and casted in the ED. She is in acceptable alignment and is managed non-operatively. At her 6-week follow up she cannot actively extend her thumb. What is the etiology of this new finding and how should it be managed? Missed carpal tunnel syndrome, nerve autograft **Extensor pollicis longus tendon rupture, extensor indicis proprius tendon transfer** Tendon adhesions, manipulation under anesthesia and aggressive physical therapy Routine post-cast stiffness, physical therapy only
13. Which of the following factors has been found to significantly impact the DASH score in patients who undergo non-operative management of DRFs? Occupation **Depression** Hand dominance Pain-escaping behavior	14. While reducing a grossly unstable DRF, you find that supinating the arm lessens re-displacement of your reduction. In the below figure, identify and name the muscle that was contributing to re-displacement of your reduction and which was neutralized by supination.
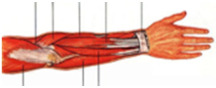
15. You are considering multi-planar external fixation versus non-operative management of a DRF in a 68-year-old male. When counseling this patient, which of the following is true regarding external fixation? Significantly better radial inclination radiographically compared to closed management Better wrist extension than closed management Improved DASH scores compared to closed management Better pinch and grip strength compared to closed management Significantly better palmar tilt compared to closed management A–C A, D, E **B, E** B, D, E	
